# The Marine Microbial Eukaryote Transcriptome Sequencing Project (MMETSP): Illuminating the Functional Diversity of Eukaryotic Life in the Oceans through Transcriptome Sequencing

**DOI:** 10.1371/journal.pbio.1001889

**Published:** 2014-06-24

**Authors:** Patrick J. Keeling, Fabien Burki, Heather M. Wilcox, Bassem Allam, Eric E. Allen, Linda A. Amaral-Zettler, E. Virginia Armbrust, John M. Archibald, Arvind K. Bharti, Callum J. Bell, Bank Beszteri, Kay D. Bidle, Connor T. Cameron, Lisa Campbell, David A. Caron, Rose Ann Cattolico, Jackie L. Collier, Kathryn Coyne, Simon K. Davy, Phillipe Deschamps, Sonya T. Dyhrman, Bente Edvardsen, Ruth D. Gates, Christopher J. Gobler, Spencer J. Greenwood, Stephanie M. Guida, Jennifer L. Jacobi, Kjetill S. Jakobsen, Erick R. James, Bethany Jenkins, Uwe John, Matthew D. Johnson, Andrew R. Juhl, Anja Kamp, Laura A. Katz, Ronald Kiene, Alexander Kudryavtsev, Brian S. Leander, Senjie Lin, Connie Lovejoy, Denis Lynn, Adrian Marchetti, George McManus, Aurora M. Nedelcu, Susanne Menden-Deuer, Cristina Miceli, Thomas Mock, Marina Montresor, Mary Ann Moran, Shauna Murray, Govind Nadathur, Satoshi Nagai, Peter B. Ngam, Brian Palenik, Jan Pawlowski, Giulio Petroni, Gwenael Piganeau, Matthew C. Posewitz, Karin Rengefors, Giovanna Romano, Mary E. Rumpho, Tatiana Rynearson, Kelly B. Schilling, Declan C. Schroeder, Alastair G. B. Simpson, Claudio H. Slamovits, David R. Smith, G. Jason Smith, Sarah R. Smith, Heidi M. Sosik, Peter Stief, Edward Theriot, Scott N. Twary, Pooja E. Umale, Daniel Vaulot, Boris Wawrik, Glen L. Wheeler, William H. Wilson, Yan Xu, Adriana Zingone, Alexandra Z. Worden

**Affiliations:** 1 Department of Botany, University of British Columbia, Vancouver, British Columbia, Canada; 2 Canadian Institute for Advanced Research, Integrated Microbial Biodiversity program, Canada; 3 Monterey Bay Aquarium Research Institute, Moss Landing, California, United States of America; 4 School of Marine and Atmospheric Sciences, Stony Brook University, Stony Brook, New York, United States of America; 5 Marine Biology Research Division, Scripps Institution of Oceanography, University of California, San Diego, La Jolla, California, United States of America; 6 The Josephine Bay Paul Center for Comparative Molecular Biology and Evolution, Marine Biological Laboratory, Woods Hole, Massachusetts, United States of America; 7 Department of Geological Sciences, Brown University, Providence, Rhode Island, United States of America; 8 School of Oceanography, University of Washington, Seattle, Washington, United States of America; 9 Department of Biochemistry & Molecular Biology, Dalhousie University, Halifax, Nova Scotia, Canada; 10 National Center for Genome Resources, Santa Fe, New Mexico, United States of America; 11 Alfred Wegener Institute Helmholtz Center for Polar and Marine Research, Bremerhaven, Germany; 12 Institute of Marine and Coastal Science, Rutgers University, New Brunswick, New Jersey, United States of America; 13 Department of Oceanography, Department of Biology, Texas A&M University, College Station, Texas, United States of America; 14 Department of Biology, University of Southern California, Los Angeles, California, United States of America; 15 Department of Biology, University of Washington, Seattle, Washington, United States of America; 16 University of Delaware, School of Marine Science and Policy, College of Earth, Ocean, and Environment, Lewes, Delaware, United States of America; 17 School of Biological Sciences, Victoria University of Wellington, Wellington, New Zealand; 18 Unité d'Ecologie, Systematique et Evolution, CNRS UMR8079, Université Paris-Sud, Orsay, France; 19 Department of Earth and Environmental Sciences and the Lamont-Doherty Earth Observatory, Columbia University, New York, New York, United States of America; 20 Department of Biosciences, University of Oslo, Oslo, Norway; 21 Hawaii Institute of Marine Biology, University of Hawaii, Hawaii, United States of America; 22 Department of Biomedical Sciences and AVC Lobster Science Centre, Atlantic Veterinary College, University of Prince Edward Island, Charlottetown, Prince Edward Island, Canada; 23 Department of Cell and Molecular Biology, The University of Rhode Island, Kingston, Rhode Island, United States of America; 24 Graduate School of Oceanography, University of Rhode Island, Narragansett, Rhode Island, United States of America; 25 Woods Hole Oceanographic Institution, Woods Hole, Massachusetts, United States of America; 26 Max Planck Institute for Marine Microbiology, Bremen, Germany; 27 Jacobs University Bremen, Molecular Life Science Research Center, Bremen, Germany; 28 Department of Biological Sciences, Smith College, Northampton, Massachusetts, United States of America; 29 University of South Alabama, Dauphin Island Sea Lab, Mobile, Alabama, United States of America; 30 Department of Invertebrate Zoology, Saint-Petersburg State University, Saint-Petersburg, Russia; 31 Department of Genetics and Evolution, University of Geneva, Geneva, Switzerland; 32 Department of Marine Sciences, University of Connecticut, Groton, Connecticut, United States of America; 33 Département de Biologie, Université Laval, Québec, Canada; 34 Department of Integrative Biology, University of Guelph, Guelph, Ontario, Canada; 35 Department of Zoology, University of British Columbia, Vancouver, British Columbia, Canada; 36 Department of Marine Sciences, University of North Carolina, Chapel Hill, North Carolina, United States of America; 37 University of New Brunswick, Department of Biology, Fredericton, New Brusnswick, Canada; 38 School of Biosciences and Biotechnology, University of Camerino, Camerino, Italy; 39 School of Environmental Sciences, University of East Anglia, Norwich, United Kingdom; 40 Stazione Zoologica Anton Dohrn, Naples, Italy; 41 Department of Marine Sciences, University of Georgia, Athens, Georgia, United States of America; 42 Plant Functional Biology and Climate Change Cluster (C3), University of Technology, Sydney, Australia; 43 Department of Marine Sciences, University of Puerto Rico, Mayaguez, Puerto Rico, United States of America; 44 National Research Institute of Fisheries Science, Kanagawa, Japan; 45 Department of Biology, University of Pisa, Pisa, Italy; 46 CNRS, UMR 7232, BIOM, Observatoire Océanologique, Banyuls-sur-Mer, France; 47 Sorbonne Universités, UPMC Univ Paris 06, UMR 7232, BIOM, Banyuls-sur-Mer, France; 48 Department of Chemistry and Geochemistry, Colorado School of Mines, Golden, Colorado, United States of America; 49 Department of Biology, Lund University, Lund, Sweden; 50 Department of Molecular and Cell Biology, University of Connecticut, Storrs, Connecticut, United States of America; 51 The Marine Biological Association of the United Kingdom, Plymouth, United Kingdom; 52 Department of Biology, Dalhousie University, Halifax, Nova Scotia, Canada; 53 University of Western Ontario, London, Ontario, Canada; 54 Moss Landing Marine Laboratories, Moss Landing, California, United States of America; 55 Section of Integrative Biology, University of Texas, Austin, Texas, United States of America; 56 Los Alamos National Laboratory, Biosciences, Los Alamos, New Mexico, United States of America; 57 UMR714, CNRS and UPMC (Paris-06), Station Biologique, Roscoff, France; 58 Department of Microbiology and Plant Biology, University of Oklahoma, Norman, Oklahoma, United States of America; 59 Plymouth Marine Laboratory, Plymouth, United Kingdom; 60 NCMA, Bigelow Laboratory for Ocean Sciences, East Boothbay, Maine, United States of America; 61 Princeton University, Princeton, New Jersey, United States of America; PLOS, United Kingdom

## Abstract

Current sampling of genomic sequence data from eukaryotes is relatively poor, biased, and inadequate to address important questions about their biology, evolution, and ecology; this Community Page describes a resource of 700 transcriptomes from marine microbial eukaryotes to help understand their role in the world's oceans.

Microbial ecology is plagued by problems of an abstract nature. Cell sizes are so small and population sizes so large that both are virtually incomprehensible. Niches are so far from our everyday experience as to make their very definition elusive. Organisms that may be abundant and critical to our survival are little understood, seldom described and/or cultured, and sometimes yet to be even seen. One way to confront these problems is to use data of an even more abstract nature: molecular sequence data. Massive environmental nucleic acid sequencing, such as metagenomics or metatranscriptomics, promises functional analysis of microbial communities as a whole, without prior knowledge of which organisms are in the environment or exactly how they are interacting. But sequence-based ecological studies nearly always use a comparative approach, and that requires relevant reference sequences, which are an extremely limited resource when it comes to microbial eukaryotes [1].

In practice, this means sequence databases need to be populated with enormous quantities of data for which we have some certainties about the source. Most important is the taxonomic identity of the organism from which a sequence is derived and as much functional identification of the encoded proteins as possible. In an ideal world, such information would be available as a large set of complete, well-curated, and annotated genomes for all the major organisms from the environment in question. Reality substantially diverges from this ideal, but at least for bacterial molecular ecology, there is a database consisting of thousands of complete genomes from a wide range of taxa, supplemented by a phylogeny-driven approach to diversifying genomics [2]. For eukaryotes, the number of available genomes is far, far fewer, and we have relied much more heavily on random growth of sequence databases [3],[4], raising the question as to whether this is fit for purpose.

## The Wrong Biases

Compared with those of prokaryotes, nuclear genomes are large and disproportionately difficult to analyze, and this means that eukaryotic genomics have been even more strongly affected by “prioritization.” This results in acute taxonomic biases in the nuclear genomes chosen for sequencing, with a large proportion of them being derived from organisms of particular biomedical or biotechnological significance. Specifically, the great majority of nuclear genomes come from animals, fungi, and plants, and from parasites that infect animals [3],[4]. For marine systems, this makes for a weak reference database, because these organisms are collectively a poor representation of eukaryotic life in the seas. Indeed, the marine organisms that maintain Earth's atmosphere, fuel the world's fisheries, and sustain the historical (pre-anthropogenic) global carbon cycle, as well as major chemical and nutrient cycles in the ocean, fall outside these groups. The lack of appropriate reference sequences risks erroneous conclusions as we compare marine ecological sequence data to references too phylogenetically distant and, therefore, too biologically different.

Each sequenced genome of an aquatic unicellular eukaryote has provided a bevy of new and unexpected insights (e.g., [5]–[13]). However, because nuclear genomes can be difficult to sequence and assemble, and gene modeling is not always straightforward, our immediate needs require an alternative way to generate a reference database, the most obvious being transcriptomics [1]. Large-scale sequencing of an organism's mRNA allows the rapid and efficient characterization of expressed genes without spending sequencing resources on the large intergenic regions, introns, and repetitive DNA so common to eukaryotes, while at the same time eliminating many problems with assembly as well as gene prediction and modeling. As a first step, transcriptomes from pure cultures are suitable building blocks to begin to assemble reference databases for eukaryotic microbial ecology. This approach generates a large number of coding sequences (in the form of assembled contigs) from a known organism.

The availability of transcriptomic data from an organism should not be viewed, however, as a substitute for sequencing its genome. The two approaches have different strengths and weaknesses and are better viewed as complementary rather than “either/or.” Indeed, nuclear genome sequencing generally requires substantial transcript sequencing to inform gene prediction algorithms. As sequencing and computational methods grow increasingly powerful, many of the challenges to genome sequencing are being reduced. Nevertheless, until more genomes are available, transcriptomes from a sufficient number of representative species from a given environment could provide a valuable benchmark against which environmental data can be analyzed.

## MMETSP—The Right Stuff

**Figure 1:**
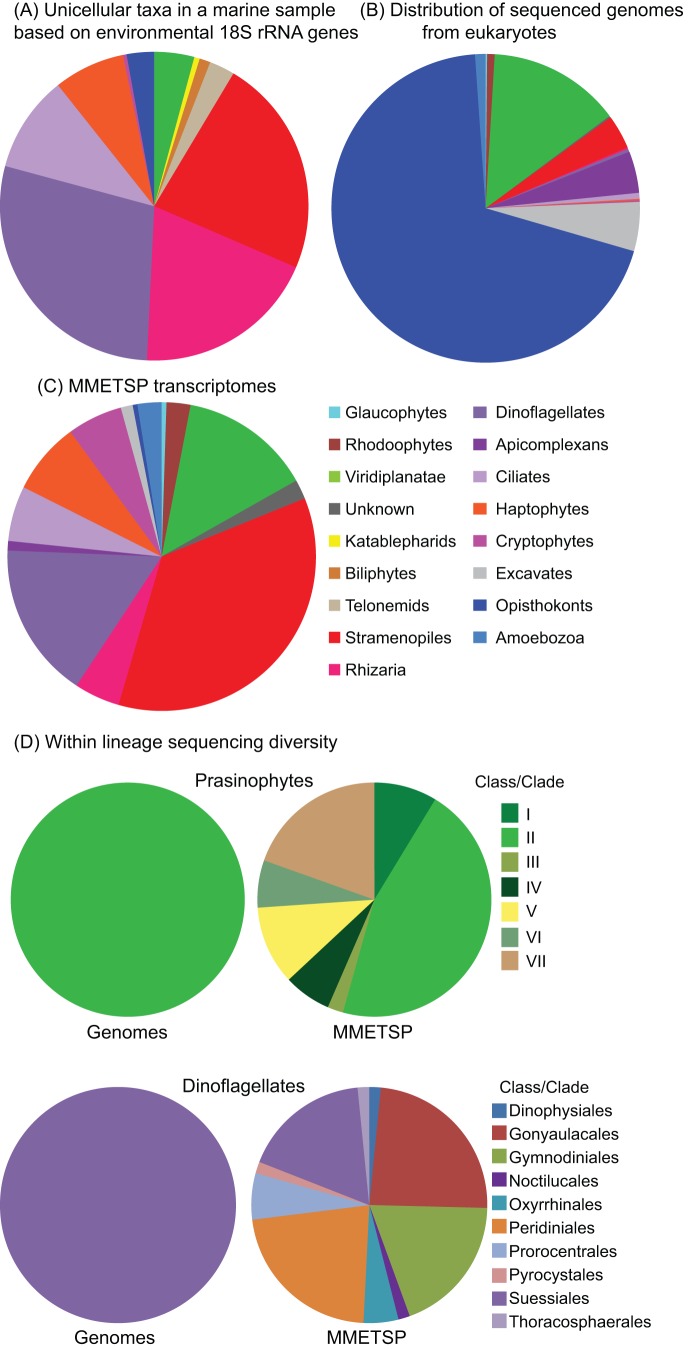
Comparing the diversity of microbial eukaryotes at one marine site with that represented in genome data and the MMETSP project. (A) Taxon assignments for 930 Small Subunit (SSU) rRNA gene sequences from environmental clone libraries built using DNA from three size fractions in sunlit surface waters of the North Pacific Ocean. Four hundred and five sequences corresponding to Syndiniales (nonphotosynthetic members of the dinoflagellate lineage, often referred to as MALV1 and MALV2) were excluded for visualization purposes. Syndiniales are not represented in any complete genome data or the MMETSP, and the vast majority are only known as sequences from uncultivated taxa that often dominate clone libraries [22],[31]. Filter size fractions were 0.1 to <0.8 µm, 0.8 to <3 µm, and 3 to <20 µm. This graph is only intended to give a snapshot of one marine sample; relative distributions vary based on distance from shore and depth, and several studies provide more detailed reviews of available SSU rRNA gene sequence surveys, see e.g., [21],[32]. (B) Taxonomic diversity of eukaryotes with complete genome sequences, as summarized in the Genomes Online Database (GOLD: http://genomesonline.org). Note that multicellular organisms are included (unlike in A or C); animals, land plants, and multicellular rhodophytes are included in the opisthokont, viridiplantae, and rhodophyte categories, respectively. (C) Taxon breakdown of the MMETSP sequencing project, collapsed at the strain level (for some strains, cells were grown under multiple conditions and these have been counted only once). (D) Comparison of currently available complete genomes and MMETSP transcriptomes by Class for two diverse and well-studied groups of algae, prasinophytes [14] and dinoflagellates [15],[16]. For both lineages, genomes are broken down by Class on the left and MMETSP transcriptomes on the right.

The Marine Microbial Eukaryotic Transcriptome Sequencing Project, or MMETSP, aims to provide a significant foothold for integrating microbial eukaryotes into marine ecology by creating over 650 assembled, functionally annotated, and publicly available transcriptomes. These transcriptomes largely come from some of the more abundant and ecologically significant microbial eukaryotes in the oceans. The choice of species, strain, and physiological condition was based on a grassroots nomination process, where researchers working in the field nominated projects based on phylogeny, environmental and ecological importance, physiological impact, and other diverse criteria. The data have been assembled and annotated by homology with existing databases (see Text S1), providing baseline information on gene function. Because the majority of transcriptomes were sequenced from cultured species, they are also taxonomically well defined. Most organisms are available from public culture collections and, therefore, can be further investigated based on hypotheses derived from the transcriptome data. The project as a whole will go a substantial distance towards fulfilling the two criteria for relevant reference sequences noted above. This is not to say these data solve all our problems: new biases have been introduced (see below), and Illumina-based transcriptomes can be challenging to assemble and work with. In addition, there is an apparently universal problem of low levels of contamination—some from other species living with the target organism in culture, others possibly from the process of library construction and sequencing. Importantly, however, the taxa from which these data are derived on aggregate conform much more closely to our understanding of marine eukaryotic diversity from sequence surveys than do the current reference databases, which are the result of ad hoc sequencing priorities that do not fit those of marine ecology (Figure 1A–1C). Indeed, digging deeper into the taxonomy of the more abundant and generally better-studied groups such as prasinophytes [14] and dinoflagellates [15],[16] shows this to be true at multiple levels (Figure 1D).

For the MMETSP data to achieve maximum impact, the transcriptomes have been made readily available through the CAMERA [17] Data Distribution Center (http://camera.crbs.ucsd.edu/mmetsp/), in which all MMETSP data have been automatically deposited. In addition, all data is in the Sequence Read Archive (SRA) under BioProject PRJNA231566, giving access to the raw trace data through GenBank. Given that library construction is not as robustly consistent as one might hope and that Illumina RNAseq assembly (in the absence of a sequenced genome) is not a completely solved problem, it is helpful that all of this work occurred at a single sequencing center where the protocols used for the >650 transcriptomes were similar (see Text S1 for a full description of methods). This approach not only broadened the types of participating labs (i.e., not just those with experience in genomics) but also maximized comparability of the datasets without the user feeling obliged to reassemble contigs, or to re-predict protein sequences for consistency. At the same time, the availability through the SRA allows for re-analysis of particular datasets.

## More Than a Reference Database

**Figure 2:**
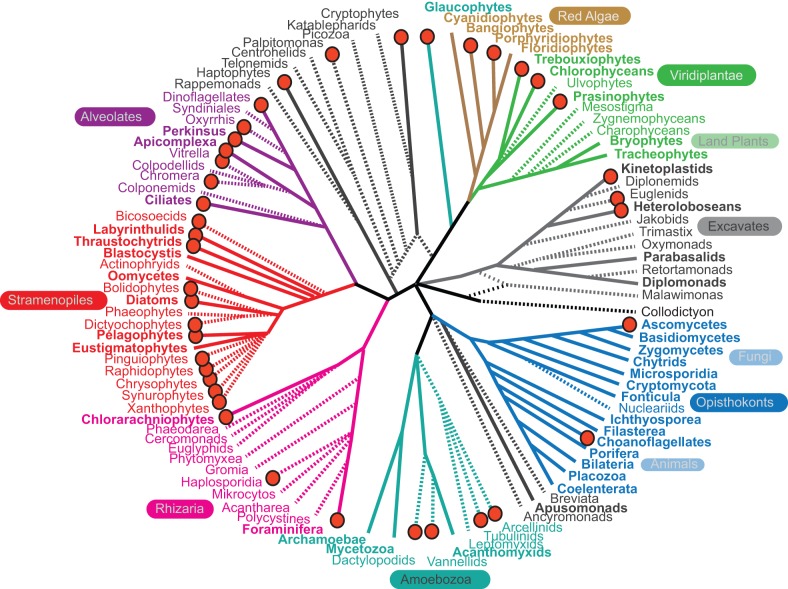
A schematic of the major lineages in the eukaryotic tree of life, showing the relationships between lineages for which genomic resources are currently available and those that have been targeted by the MMETSP. Lineages with complete genomes according to the GOLD database, as summarized in [3], are indicated by a solid line leading to that group, whereas lineages with no complete genome are represented by a dashed line. Lineages where at least one MMETSP transcriptome is complete or underway are indicated with a red dot by the name. Major lineages discussed in the text have been named and color-coded, but for clarity, some major lineages have not been labeled.

The more than 650 transcriptomes will have far-reaching impact beyond the field of marine science. The diversity of taxa represented in the database is impressive, even when held up to the enormous diversity of microbial eukaryotes as a whole (Figure 2). In some cases, these data provide the first glimpse of the genome of an important group of microbial eukaryotes, such as parasitic haplosporidia, several amoebozoans, and the enigmatic heterotrophic flagellate *Palpitomonas*. In other cases, they provide genomic data from a diverse selection of taxa within a lineage where only sparse genomic data previously existed from a few distant relatives (such as the ciliates [18]–[20]). Experience has shown that such data can transform our understanding of the basic biology and function of these organisms. In the past, we have described a protistan lineage for which there is a single genome sequence as being “well studied.” Thus, even for those that are comparatively “well studied,” the MMETSP data facilitates new directions. It opens the door to comparative genomics within lineages and between related lineages in major protistan groups, including foraminifera, cryptophytes, and several groups of red algae and stramenopiles. Digging further, other cases will allow us to ask population genomic-level questions by providing data from multiple strains of a single species (or even asking whether the “multiple strains” do indeed belong to the same species!). Examining the diversity between sister species or members of the same species can help identify functionally important genes, genes under selection, recent gene family expansions and contractions, or other significant changes like horizontal gene transfer—of course, with recognition that absence from a given transcriptome assembly does not necessarily represent absence from the genome. In other cases, the same isolate has been analyzed under different physiological conditions to develop testable hypotheses on environmental controls. For example, it should be possible to gain first molecular insights into how photosynthetic algae alter their immediate surroundings, the so-called phycosphere [21], by comparing sequences from the luminescent dinoflagellate *Lingulodinium polyedrum* that is co-cultured with different bacteria, or cultured on its own. Likewise, growth controls and aspects of niche differentiation should become clearer for many major phytoplankton groups.

## A Fast Start and a Long Way to Go

The MMETSP is a significant step in recognizing that purpose-built reference databases from ecologically key biomes are essential for all domains of life. Nevertheless, it is only the beginning, and important biases remain that should be addressed. The MMETSP relies primarily on cultured organisms, and this introduces a different set of biases, most obviously, favoring organisms that are photosynthetic. Eukaryotic heterotrophs have critical ecological roles but are under-represented. Indeed, the natural diversity of eukaryotic heterotrophs is huge in general (Figure 1A), and the four most commonly recovered sequences retrieved in environmental surveys of marine samples worldwide correspond to lineages for which most members are uncultivated (e.g.,Marine Stramenopiles (MAST) and Marine Alveolates (MALV) [22]–[24]). These are probably heterotrophs, but we lack a solid biological definition for most of these cells and have become adroit at ignoring heterotrophs in general. Similarly, organisms from the open ocean are underrepresented. Culture-independent methods for generating transcriptomes and genomes and, in some cases, transcriptomes and genomes from single cells will be essential to moving beyond this problem. Methodologies for population [25]–[27] and single-cell genomics and transcriptomics are advancing rapidly [4],[28]–[30], transitioning from technological feats to something we should expect to work routinely. This transition holds great promise for filling the rather substantial gap in our knowledge imposed by uncultivated protists, as well as allowing us to carry out condition-specific analyses of expressed genes in difficult-to-work-with systems. The MMETSP program foreshadows this development by sequencing a small set of culture-independent samples.

The MMETSP dataset serves as an example of how purpose-built reference databases focused on a particular niche or environment can be established relatively quickly and efficiently. This database will allow us to address eukaryotic sequences from nature in a robust manner for the first time. Because the strength of the MMETSP project is precisely its focus on the marine environment, it will not serve as a universal database of eukaryotic diversity that can be easily applied to other environments. While the taxonomic diversity included in the project is amazing (Figure 2), it is also immediately clear that many major groups of eukaryotes are not covered by MMETSP transcriptomes. In some cases, this is because these lineages are not abundant in the oceans (e.g., many excavates), but in others it is simply because members of the lineage are difficult to cultivate and are generally poorly represented in molecular data (e.g., most rhizarians), even if they are abundant and important in the ocean. For other major environments (e.g., freshwater, soil) similar databases could be developed in a focused manner, but all such efforts rely on a detailed knowledge of what lives in that environment, which is not always adequate. To remedy these gaps in our knowledge, we advocate a taxonomy-based approach similar to the Genomic Encyclopedia of Bacteria and Archaea (www.jgi.doe.gov/programs/GEBA/) [2],[4]. This undertaking will require a focus on developing the necessary tools for gaining access to the transcriptomes and genomes of uncultivated organisms and would represent a major advance for all aspects of the study of microbial eukaryotes. We look forward to the many creative analyses and results enabled by the MMETSP and the minds of the broader scientific community; the new insights to be gained in ecology, physiology, and evolution of unicellular eukaryotes will significantly advance understanding of marine ecosystems and eukaryotic microbial biology as a whole. The MMETSP illustrates the power behind such a community activity and bodes well for a future Genomic Encyclopedia of Microbial Eukaryotes.

## Supporting Information

Text S1 The supplementary methods file contains a referenced description of the standardized methods used for transcriptome sequencing, assembly, and analysis used for all MMETSP projects.
(DOC)
