# Supplementary material for: The Marine Microbial Eukaryote Transcriptome Sequencing Project (MMETSP): Illuminating the Functional Diversity of Eukaryotic Life in the Oceans through Transcriptome Sequencing
Source: PLoS Biol. 2014 Jun 24;12(6):e1001889. doi: 10.1371/journal.pbio.1001889 (PMC4068987; doi:10.1371/journal.pbio.1001889)
Supplement: Text S1 — The supplementary methods file contains a referenced description of the standardized methods used for transcriptome sequencing, assembly, and analysis used for all MMETSP projects. (DOC) [file pbio.1001889.s001.doc]

Supplementary Materials and methods: Protists were grown and RNA extracted according to procedures used in each contributing lab, and bacteria are common components of many such cultures. Total RNA samples were quality checked (QC) using Qubit for quantity as well as with Bioanalyzer for quality.

Paired-ended Illumina sequencing (Illumina, San Diego CA, USA) was performed at the National Center for Genome Resources, NCGR (Santa Fe NM, USA). Libraries were made from 500 ng to 2 µg RNA using the TruSeq RNA Sample Preparation Kit (Illumina). Pre- and post- PCR steps were carried out in physically separate rooms. A small number of laboratories sent cDNA directly. Average library fragment sizes ranged from 250 to 350 base pairs (bp). Over 2 Gb of sequence was generated for each library on a HiSeq 2000 in paired-end 50 bp configuration (a small number of samples were paired-end 100 bp). Transcriptome assembly was carried out using NCGR’s BPA1.0 (Batch Parallel Assembly v. 1.0) and BPA2.0 pipelines, as the methods were refined during the 2 year effort. The primary focus of BPA2.0 development was to reduce sequence redundancy in the transcriptome assemblies and the code is available on GitHub (https://github.com/ncgr/rbpa).

Initial steps for BPA1.0, and BPA2.0 were identical: sequence reads were preprocessed using SGA [[1](#ENREF_1)] preprocess for quality trimming (swinging average) at Q15, those less than half the initial read length after trimming were discarded. The remaining reads were assembled with ABySS [[2](#ENREF_2)] v. 1.3.0, using 20 unique kmers between k=26 and k=50 (between k=51 and k=100 for paired-end 100 bp). In both cases, ABySS was run using a minimum kmer coverage of 5, bubble popping at >0.9 branch identity, however in BPA1.0 the scaffolding flag was enabled to maintain contiguity for divergent branching where as in BPA2.0 the scaffolding flag was disabled to avoid over reduction of divergent regions. From here, in BPA1.0 paired-end scaffolding was performed on each kmer. Sequence read pairing was used in GapCloser, part of SOAP de novo package v. 1.10 [[3](#ENREF_3)], to walk in on gaps created during scaffolding in each individual kmer assembly. Contigs from all gap-closed kmer assemblies were combined. The overlap layout consensus assembler MIRA [[4](#ENREF_4)] was used to identify minimum 100 bp overlaps between the contigs and assemble larger contigs, while collapsing redundancies. BWA [[5](#ENREF_5)] was used to align sequence reads back to the contigs. Alignments were processed by SAMtools mpileup ([http://samtools](http://samtools/).sourceforge.net) to generate consensus nucleotides at positions where IUPAC bases were introduced by MIRA [[4](#ENREF_4)], and read composition showed a predominance of a single base. For BPA2.0, unitigs from all kmer assemblies were combined and redundancies were removed using CD-HIT-EST [[6](#ENREF_6)] with a clustering threshold of 0.98 identity. The OLC (overlap layout consensus) assembler CAP3 [[7](#ENREF_7)] was then used to identify minimum 100 bp overlaps between the resultant contigs and assemble larger sequence. The resulting contigs were paired-end scaffolded using ABySS [[2](#ENREF_2)]. Sequence read pairing was used in GapCloser, part of the SOAP de novo package v. 1.10 [[3](#ENREF_3)], to walk in on gaps created during scaffolding. Redundant sequences were again removed using CD-HIT-EST [[6](#ENREF_6)] at a clustering threshold of 0.98 identity. For both pipeline versions consensus contigs <150 bp were discarded.

Slight modifications were made for samples submitted as cDNA. The poly-A selection step of the TruSeq RNA Sample Preparation protocol was skipped during library preparation. If adapters were used for cDNA isolation, those adapters were trimmed from the sequence reads using FASTX Clipper (<http://hannonlab.cshl.edu/fastx_toolkit>) prior to assembly.

ESTScan v. 3.0.3 [[8](#ENREF_8),[9](#ENREF_9)] was used to create peptide prediction files after training with RefSeq mRNA entries under the Bacillariophyta classification. Peptides <30 aa were discarded. BLASTP [[10](#ENREF_10)] was used to generate hits to the UniProtKB/Swiss-Prot database (http://www.uniprot.org/downloads). HMMER3 [[11](#ENREF_11)] was used for functional classification against Pfam-A [[12](#ENREF_12)], SUPERFAMILY [[13](#ENREF_13)], and TIGRFAM [[14](#ENREF_14)].

Transcriptome taxon distributions shown here are based on a compilation of data provided on the MMETSP website as of October 2013 at which time 685 individual transcriptomes were listed. For charts, transcriptome counts were collapsed at the strain level prior to computation of percentage of the total. 18S rRNA gene data used in Figure 1A comes from Line 67 station 67-155 collected in October 2007 (details of the site are provided in [[15](#ENREF_15)]). Genes were cloned from PCR products from each size fraction and from two depths (10 m and 76 m), the latter corresponding to the deep chlorophyll maximum. Environmental clones were BLASTed against the PR2 database and parsed according to the ID of the best hit.

Supplementary references

1. Simpson JT, Durbin R (2012) Efficient de novo assembly of large genomes using compressed data structures. Genome Res 22: 549-556.

2. Simpson JT, Wong K, Jackman SD, Schein JE, Jones SJ, et al. (2009) ABySS: a parallel assembler for short read sequence data. Genome Res 19: 1117-1123.

3. Li R, Li Y, Kristiansen K, Wang J (2008) SOAP: short oligonucleotide alignment program. Bioinformatics 24: 713-714.

4. Chevreux B, Pfisterer T, Drescher B, Driesel AJ, Muller WE, et al. (2004) Using the miraEST assembler for reliable and automated mRNA transcript assembly and SNP detection in sequenced ESTs. Genome Res 14: 1147-1159.

5. Li H, Durbin R (2009) Fast and accurate short read alignment with Burrows-Wheeler transform. Bioinformatics 25: 1754-1760.

6. Li W, Godzik A (2006) Cd-hit: a fast program for clustering and comparing large sets of protein or nucleotide sequences. Bioinformatics 22: 1658-1659.

7. Huang X, Madan A (1999) CAP3: A DNA sequence assembly program. Genome Res 9: 868-877.

8. Iseli C, Jongeneel CV, Bucher P (1999) ESTScan: a program for detecting, evaluating, and reconstructing potential coding regions in EST sequences. Proc Int Conf Intell Syst Mol Biol: 138-148.

9. Lottaz C, Iseli C, Jongeneel CV, Bucher P (2003) Modeling sequencing errors by combining Hidden Markov models. Bioinformatics 19 Suppl 2: ii103-112.

10. Altschul SF, Gish W, Miller W, Myers EW, Lipman DJ (1990) Basic local alignment search tool. Journal of Molecular Biology 215: 403-410.

11. Zhang Z, Wood WI (2003) A profile hidden Markov model for signal peptides generated by HMMER. Bioinformatics 19: 307-308.

12. Finn RD, Mistry J, Tate J, Coggill P, Heger A, et al. (2010) The Pfam protein families database. Nucleic Acids Res 38: D211-222.

13. Gough J, Karplus K, Hughey R, Chothia C (2001) Assignment of homology to genome sequences using a library of hidden Markov models that represent all proteins of known structure. J Mol Biol 313: 903-919.

14. Haft DH, Loftus BJ, Richardson DL, Yang F, Eisen JA, et al. (2001) TIGRFAMs: a protein family resource for the functional identification of proteins. Nucleic Acids Res 29: 41-43.

15. Monier A, Welsh RM, Gentemann C, Weinstock G, Sodergren E, et al. (2012) Phosphate transporters in marine phytoplankton and their viruses: cross-domain commonalities in viral-host gene exchanges. Environmental Microbiology 14: 162-176.
